# Supplementary material for: Probing nanofriction and Aubry-type signatures in a finite self-organized system
Source: Nat Commun. 2017 May 15;8:15364. doi: 10.1038/ncomms15364 (PMC5440669; doi:10.1038/ncomms15364)
Supplement: Supplementary Information — Supplementary Figures and Supplementary Notes [file ncomms15364-s1.pdf]

Supplementary Part

Supplementary Note 1: Determination of characteristic frequencies of the inter- and intra- row interactions

We define the corrugation parameter as $\eta = \omega_{\mathrm{pinning}}^2/\omega_{\mathrm{natural}}^2$, where $\omega_{\mathrm{pinning}}$ is the frequency of vibration of an ion in a potential generated by an adjacent row of ions and $\omega_{\mathrm{natural}}$ is the frequency of vibration in a potential generated by ions within a row. Here, we describe the algorithm used to compute these frequencies.

For a given control parameter $\alpha = \omega_{\mathrm{x}}/\omega_{\mathrm{z}}$, the stable equilibrium ion positions in a regular zigzag are determined numerically using gradient descent method. U_{inter} is defined as the potential energy of an ion in the chain, which is generated by all ions in the opposing row and the harmonic trapping potential. To obtain the pinning frequency $\omega_{\mathrm{pinning}}$, we Taylor expand U_{inter} in small axial displacements q around the location of the ion that is nearest to the crystal centre $\mathbf{r}_0 = (z_0, x_0)$. The pinning frequency is given by $\omega_{\mathrm{pinning}}^2 = \frac{1}{m}\frac{\partial^2 U_{\mathrm{inter}}}{\partial q}\Big|_{\mathbf{r}_0}$, where m is the mass of the ion.

U_{intra} is defined as the potential energy of an ion in a chain, which is generated by all remaining ions in the same row. To obtain $\omega_{\mathrm{natural}}$, we Taylor expand U_{intra} in small axial displacements q about the location of the ion of interest $\mathbf{r}_0 = (z_0, x_0)$. The natural frequency is given by $\omega_{\mathrm{natural}}^2 = \frac{1}{m}\frac{\partial^2 U_{\mathrm{intra}}}{\partial q}\Big|_{\mathbf{r}_0}$.

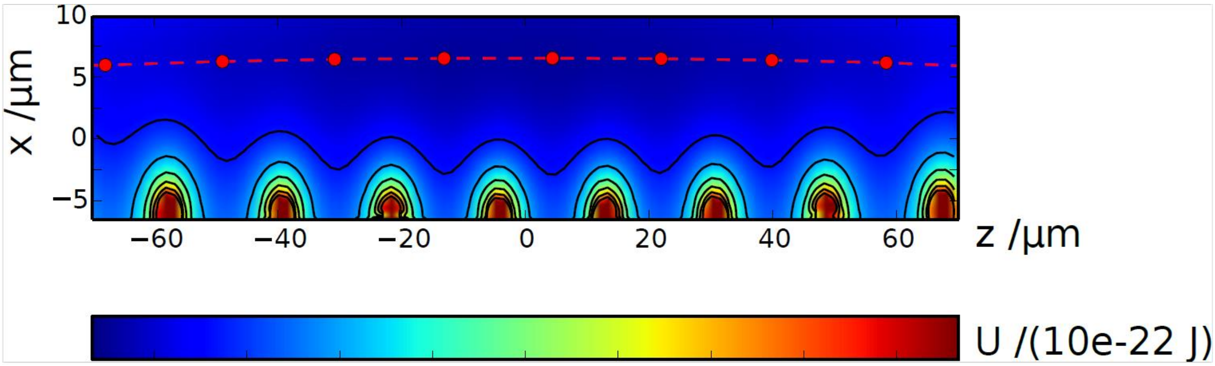

Supplementary Figure 1: Corrugation Potential of an ion chain. *Potential energy surface generated by the bottom row of a harmonically trapped Coulomb crystal of 30 ions in a zigzag configuration. The secular frequencies using the calculation were $\omega_{\mathrm{x}} = 160.1$ kHz and $\omega_{\mathrm{z}} = 24.6$ kHz. The red dots indicate the positions of ions in the top row.*

We compare the calculated corrugation parameter η to the given trap frequency ratio α numerically. In an interval $\delta\alpha = 0.2$ around the critical point $\alpha_{\mathrm{c}} = 6.41$, η scales linearly with α, see Fig. S2.

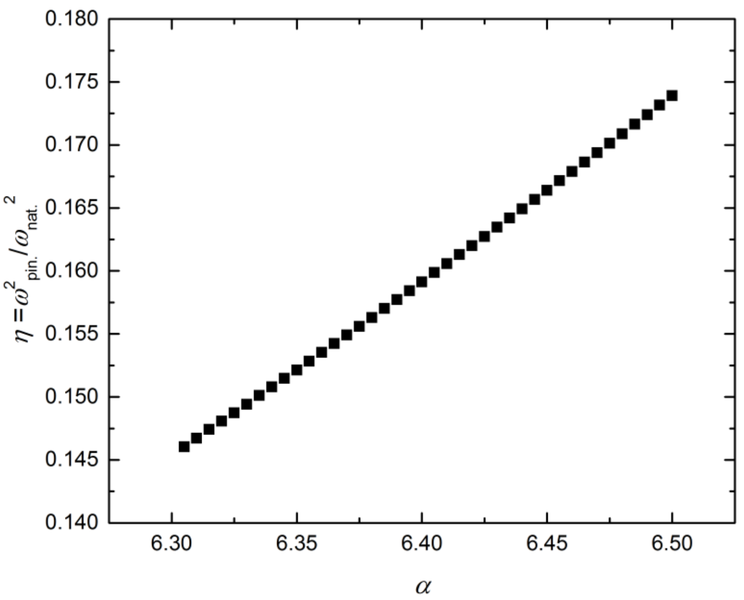

Supplementary Figure 2: Corrugation parameter. *Dependence of corrugation parameter η on trap frequency ratio α near critical point.*

Supplementary Note 2: Prandtl-Tomlinson model – critical force in commensurate crystal

In this section, we describe the method used to estimate the minimal force needed to translate two commensurate ion rows by one lattice site with respect to one another.

When the two lattices are commensurate than the particles of one row slide coherently with respect to one another. For this reason to obtain the critical sliding force F_{S}, it is sufficient to evaluate the minimal force needed to move single particle by a lattice site in a Prandtl-Tomlinson (PT) model. The potential energy of PT model is

$$V_{\text{PT}}(z) = V(z) + Fz = \frac{U_0}{2}\left(1 + \cos\frac{2\pi z}{a}\right) + Fz,$$

where z is the position of the particle and F is the externally applied force. This equation is the classic tilted washboard potential. The particle slides continuously along the substrate potential if $F > U_0\pi/a$. Thus in the case of two perfectly commensurate rows, the minimal force needed to translate one row by a lattice site is $F_{\text{S}} = U_0\pi/a$.

To map the Coulomb crystal consisting of two commensurate rows to the PT model the following procedure was used. First, we find the equilibrium crystal configuration in a system with periodic boundary conditions using gradient descent method for a specified value of inter ion spacing and transverse harmonic confinement. We then compute the potential energy that is generated by ions in one of the rows and the harmonic confinement, along the line connecting the ions in the opposing row. This sinusoidal potential energy function $V(z)$ corresponds to the potential energy of the periodic potential in the PT model. The minimal force needed to translate an ion by one lattice site (and hence a commensurate row) is $U_0\pi/a$, where $U_0/2$ is the amplitude of $V(z)$ and a is the period of $V(z)$.

Supplementary Figure 3:

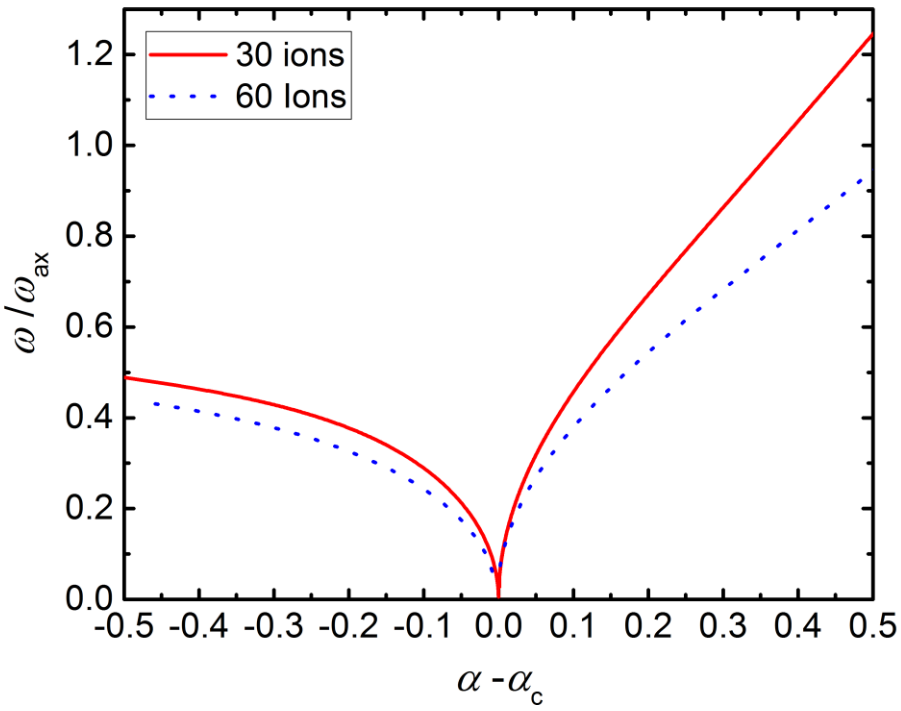

Supplementary Figure 3: Influence of the ion number on the soft mode frequency. *Soft mode frequency of an ion coulomb crystal with defect for* $N = 30$ *(red solid line) and* $N = 60$ *(blue dotted line) near the sliding to pinned transition. The transition occurs at* $\alpha_{c,30} \approx 6.41$ *for* 30 *ions and at* $\alpha_{c,60} \approx 11.09$ *for* 60 *ions. Doubling the ion number from* 30 *to* 60 *ions lowers the soft mode frequency by roughly* 15% *below the transition.*
